# Supporting athletes during a challenging situation: recommendations from a global insight of COVID-19 home-based training experience

**DOI:** 10.1186/s13102-024-00869-7

**Published:** 2024-04-15

**Authors:** Jad Adrian Washif, Florentina J. Hettinga, Achraf Ammar, Dina Christa Janse van Rensburg, Olivier Materne, Khaled Trabelsi, Mohamed Romdhani, Abdulaziz Farooq, David B. Pyne, Karim Chamari

**Affiliations:** 1 Sports Performance Division, Institut Sukan Negara Malaysia (National Sports Institute of Malaysia), Kuala Lumpur, Malaysia; 2 Department of Sport, Exercise and Rehabilitation, Northumbria University, Newcastle upon Tyne, UK; 3 Department of Training and Movement Science, Institute of Sport Science, Johannes Gutenberg- University Mainz, Mainz, Germany; 4 Research Laboratory, Molecular Bases of Human Pathology, Faculty of Medicine of Sfax, University of Sfax, LR19ES13 Sfax, Tunisia; 5 High Institute of Sport and Physical Education of Sfax, University of Sfax, Sfax, Tunisia; 6 Section Sports Medicine, Faculty of Health Sciences, University of Pretoria, Pretoria, South Africa; 7 Medical Board Member, World Netball, Manchester, UK; 8 The Glasgow Rangers Football Club Ltd, Glasgow, UK; 9 High Institute of Sport and Physical Education, University of Sfax, Sfax, Tunisia; 10 Research Laboratory: Education, Motricity, Sport and Health, EM2S, University of Sfax, LR19JS01 Sfax, Tunisia; 11 Physical Activity, Sport and Health, UR18JS01, National Observatory of Sports, Tunis, Tunisia; 12 Interdisciplinary Laboratory in Neurosciences, Physiology and Psychology: Physical Activity, Health and Learning (LINP2), Faculty of Sport Sciences, UPL, UFR STAPS, Paris Nanterre University, Nanterre, France; 13 Research Department, Aspetar, Orthopaedic and Sports Medicine Hospital, Doha, Qatar; 14 Research Institute for Sport and Exercise, University of Canberra, Canberra, Australia; 15 Higher Institute of Sport and Physical Education, ISSEP Ksar Saïd, Manouba University, Manouba, Tunisia; 16 Naufar Wellness and Recovery Center, Doha, Qatar

**Keywords:** Coaching, Coping strategies, Home training, Injury, Lockdown, Mental health, Modified-training, Training load, Sleep

## Abstract

**Background:**

For athletes, overcoming obstacles in challenging situations like pandemic home training is crucial. Strategies and approaches in this context are not well-documented. Our study aims to investigate such a scenario from a performance standpoint, based on a major global crisis: the COVID-19 pandemic and lockdown.

**Methods:**

This cross-sectional study surveyed athletes without disabilities using online questionnaires (35 languages) from May to July 2020. Questions included aspects of alternative routines, training monitoring, recovery, sleep patterns, injury occurrence/prevention based on structured answers, and an open-ended question on lockdown training experiences.

**Results:**

Of the 11,762 athletes from 142 countries, 63% were male, including at World-Class, International, National, State and Recreational levels. During lockdown, 25% athletes used innovative or modern ways to maintain or improve fitness e.g., virtual reality and tracking devices (favoring World-Class level, 30%). Many athletes, regardless of gender (43%) watched video competitions to improve/maintain their mental skills and performance [World-Class (47%) and International (51%)]. Contact frequency between athletes and their coaches was mainly at least once a week (36%), more among higher-level (World-Class/International) than lower-level athletes (27 vs. 16%). Higher-level athletes (≥ 54%) monitored training load and were assisted by their coaches (21%). During lockdown, stretching (67%) was considered one of the primary means of recovery, especially for higher-level athletes (> 70%). Compared to pre-lockdown, about two-thirds of athletes reported “normal” or “improved” sleep quality and quantity, suggesting a low sleep quality pre-lockdown. On average, 40% utilized injury prevention exercises (at least) once a week [World-Class (51%) and International (39%)]. Most injury occurrences during lockdown involved the knee (18%), ankle (16%), and back (9%). Four key themes emerged regarding lockdown experiences: remote training adaptation (e.g., shifting training focus), training creativity (e.g., using household items), performance enhancement opportunities (e.g., refocusing neglected aspects), and mental and motivation challenges.

**Conclusions:**

Both male and female athletes, particularly those of higher levels, displayed some adaptalibity during the COVID-19 lockdown, employing innovative approaches and technology for training. Many athletes implemented load monitoring, recovery, and attentive of injury prevention, while optimizing their sleep quality and quantity. Athletes demonstrated their abilities to navigate challenges, and utilized different coping strategies in response to the lockdown’s constraints.

## Introduction

At the onset of the COVID-19 pandemic, widespread lockdowns were implemented globally in early 2020. As a result, various non-critical operations, such as educational institutions, eateries, and sports facilities, underwent prolonged suspensions [1]. Such abrupt transitions heavily affected the sporting calendar, as well as the daily athletic (training) routines of many individuals and professionals [2, 3]. In some rare cases, specialized “bubble training camps” enabled some elite athletes to continue their usual training, albeit this option was not always available [4–6].

In challenging situations such as the COVID-19 lockdown or other epidemics/pandemics, geopolitical or religious restrictions, adverse (seasonal) weather and climatic conditions, or local political/governmental impositions, athletes must adapt to limited resources, confined spaces, and the absence of traditional team/support environments. During lockdowns, athletes have to modify their training methods while adhering to restrictive policies [1]. Constraints of home workouts, e.g., usually with modified versions of exercises and household items, have both positive and negative impacts on fitness outcomes [7]. Moreover, the risk of injury increases when employing improper training, e.g., overloading, poor techniques, unsupervised, non-systematic [8], and defective exercise equipment [9]. Competitive athletes who were accustomed to specialized and intensive daily training had to regulate their routines within “severe” limitations (e.g., home training during lockdown), often with minimal recovery resources [10]; this scenario could potentially lead to possible maladaptation or even injury. These limitations inherent to home-training (COVID-19 context), can reduce training motivation [5], affecting the ability to execute post-lockdown sports-specific tasks [11], and possibly increase the post-lockdown injury risk [12, 13] upon resumption of competitions [14].

Intuitively monitoring training (albeit remotely performed online) is essential to ensure athletes maintain a balanced training regimen, including adequate rest and recovery [10]. Recovery strategies, including sleep, are vital for physical recuperation and mental health management [15, 16]. Elite athletes are not spared from experiencing psychological distress during lockdown, partly due to lockdown-induced turbulence caused by among others training interruptions and sleep disturbances [5, 17]. The prevalence of mental health challenges among these athletes was similar to, or even exceeded, that of the general population [18–21]. Collectively, these mental health issues could negatively impact athletes by affecting their performance, heightening injury risk, and extending the recovery time from physical injuries [10, 20].

Several studies have explored the training practices and associated challenges (e.g., training, mental, or sleep) faced by athletes during the COVID-19 lockdown [17, 22, 23]. However, details on alternative training, monitoring, recovery, injury/injury prevention, and sleep patterns, especially across a single, large cohort of athletes worldwide, would be useful for developing effective athlete management strategies. Notably, overcoming obstacles in challenging situations like pandemic home training is crucial. Gaining a deeper insight into these facets of performance (training, recovery, etc.) will assist coaches/scientists in determining and providing the specific support needed in such situations. Therefore, the aim of this study was to provide a detailed characterization of performance-related aspects experienced during home training among a large sample of athletes across the world during the initial global lockdowns. We employed a combination of pre-set (answer selection format) and open-ended questions to capture more nuanced athletes’ experiences, thereby enhancing our understanding of the associated challenges beyond what was typically collected using structured answers. The study provides data-driven recommendations regarding alternative training, monitoring strategy, recovery, sleep, injury prevention, mental health regulation, and training motivation, which should be useful for athletes, coaches, and scientists in similar “challenging” situations in the future.

## Methods

### Design

In the early COVID-19 pandemic context, this cross-sectional, questionnaire-based study [1, 24] was designed to capture the multifaceted challenges athletes worldwide faced due to “stay-at-home” orders. These orders led to the closure of sport facilities, and public venues, enforced social distancing, and suspended all sport events and team activities, compelling individuals to remain indoors. Data on how these restrictions impacted various aspects of athlete performance were collected through an online questionnaire.

### Participants

A sample of adult athletes (aged 18 and above) athletes was recruited between 17 May 2020 and 7 July 2020. An online questionnaire (Google Forms) was distributed to athletes of various sports and competition levels via messaging applications (e.g., WhatsApp, Telegram, Signal), social media (e.g., Twitter, Facebook, LinkedIn), sports organization, and emails. In the current study, our focus was on adult athletes (aged 18 years or above) without disabilities who had experienced a “medium-to-high” severity lockdown for at least two consecutive weeks between March and June 2020. A medium-to-high lockdown severity was deemed in place for any of the following conditions: (i) Essential movement only for supplies/groceries; (ii) Limited access to public exercise spaces, such as parks; and/or (3) Closure of training facilities at various institutions and clubs [1]. Additionally, these athletes had not missed training for more than 7 days due to illness or injury during the survey period; those who suffered severe injuries were ineligible to participate [1, 24]. The institutional review boards (e.g., Institut Sukan Negara Malaysia, the University of Melbourne, University of Cassino e Lazio Meridionale, and Qatar University) approved the study. Data from the consenting participants were analyzed anonymously.

### Questionnaires

The questionnaire examines athletes’ alternative routines, training monitoring, recovery, sleep patterns, injury occurrence, injury-prevention practice, and overall home training experience. To enhance the reliability of responses, straightforward questions were employed. The questionnaire, and its development process is provided in a previous publication [1]. In this study, however, we present a novel dataset not previously examined within the scope of our earlier studies [1, 24]. Briefly, the principal investigators first designed the questionnaire before undergoing a review by a separate research team to verify its content validity. Subsequently, a group of athletes gave input on its readability and comprehensiveness. Based on this feedback, the questionnaires underwent further refinement until a final version was reached through consensus among the entire research team. The primary survey in English was translated into 34 languages to include all major languages, with each version subject to a rigorous two-way translation process. The English survey’s test-retest reliability was rated good to excellent [1, 24].

### Statistical analysis

Data were presented using descriptive statistics such as frequencies, percentages, and mean (SD). Chi-square (χ2) test of independence (with analysis of adjusted residuals) was utilized to assess categorical variable differences. Post-hoc significance was based on adjusted residuals with Bonferroni correction and a *p* value of < 0.05 used for all statistical analyses. All statistical analysis was performed using IBM SPSS (v26.0). A difference of ≥ 10% between comparators was deemed practically meaningful [24]. For the open-ended questions, inductive analysis was applied, whereby the remarks of respondents were reviewed carefully, organized (key ideas) into categories, and then interpreted into specific themes related to lockdown experiences; adopted from [25].

## Results

### Participant’s demographic

A sample of 11,762 male (66%) and female (34%) adult athletes, mostly aged 18–29 years (67%) from 142 countries were recruited. The athletes competed in 108 different sports, and were classified in five competition levels: World-Class (13%), International (20%), National (36%), State (25%), and Recreational (6%).

### Alternative training

During lockdown, 25% of total athletes (24% male, and 26% female) used innovative or modern ways to maintain or improve their fitness. Independent of gender, the proportion of World-Class athletes (30%) was relatively higher, with State athletes being relatively lower (23%). Training included utilization of equipment and modalities such as aerobic-based facilities (e.g., indoor bike, treadmill, sky ergo), virtual reality (e.g., smart turbo trainer with Zwift and/or Strava, fitness games), data tracking devices (e.g., Strava, polars, smartwatches), modified equipment (e.g., innovative weights such as rocks and bricks, improvized rubber band, household items), video conferencing (e.g., team training, guided fitness session), and others (e.g., wearable resistance, live streams workouts, YouTube fitness videos, making use of garage). A large portion of athletes (43% of both male and females) also watched video competitions (e.g., YouTube) while trying to maintain/improve on mental skills and performance; here, the distribution of World-Class (47%) and International (51%) athletes was higher than State (37%) and Recreational (37%) ones.

### Training monitoring

**Fig. 1:**
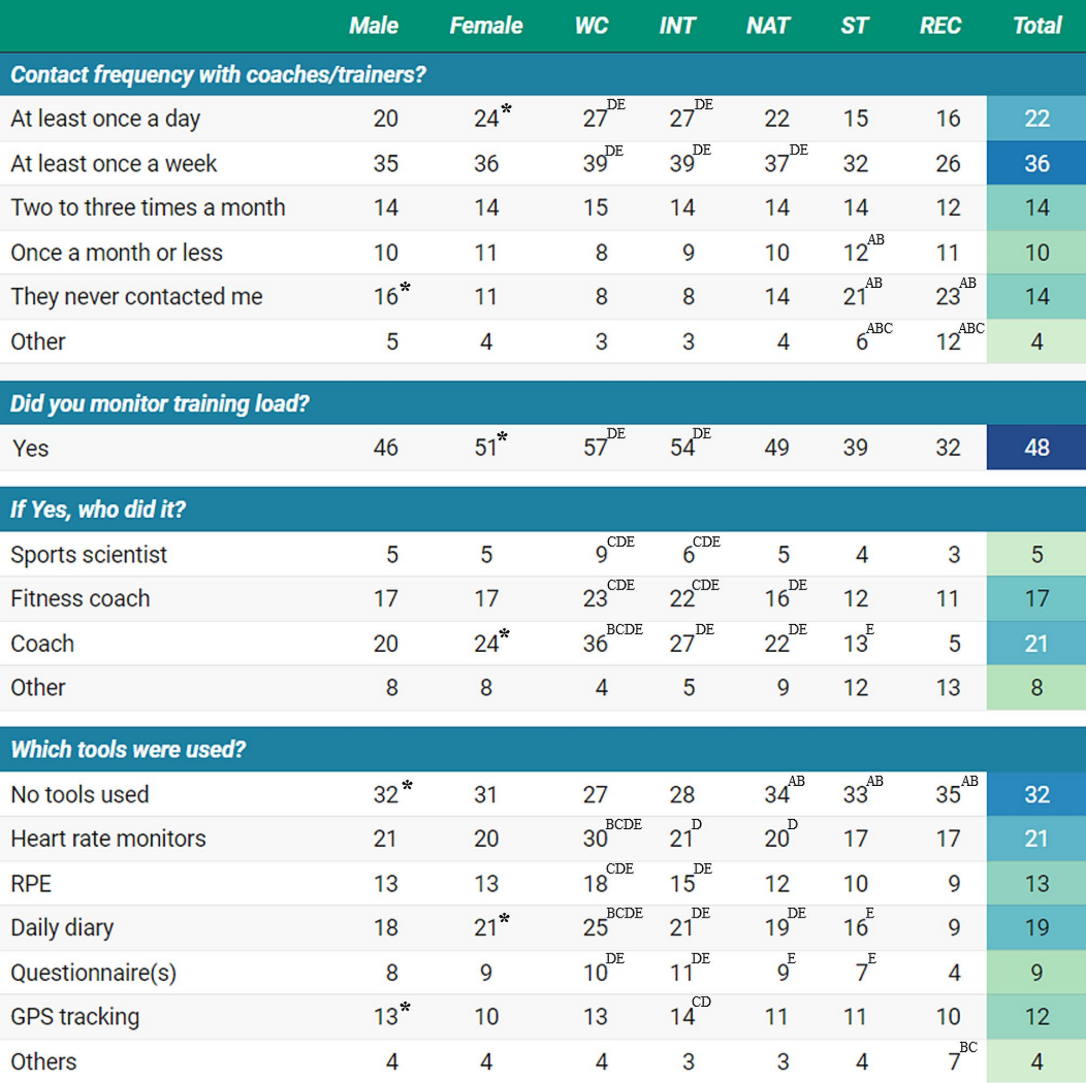
Training monitoring by gender, athlete level, and total cohort (percentage) during the COVID-19 lockdown *Note*: WC: World Class; INT: International; NAT: National; ST: State; REC: Recreational. *Significantly higher (or significant contributor to the relationship); superscript letters ^A, B,C, D,E^ represent significantly higher than World Class, International, National, State, and Recreational levels, respectively. RPE, rate of perceived exertion; GPS, Global Positioning System; %, represent ‘yes’ answer, relative to ‘no’ answer;

Across all levels, contact frequency between athletes and their coaches was mainly at least once a week (36%) or once a day (22%); here, the proportion (27%) of higher-level athletes (World-Class and International) was greater than the lower-level athletes (State 15% and Recreational 16%). More World-Class (57%) and International (54%) athletes monitored training load than State (39%) and Recreational (32%) athletes. Load monitoring was conducted mainly by coaches (21%), especially for higher-level athletes. More World-Class or International athletes used the rating of perceived exertion, daily diary, questionnaire(s), heart rate monitors, and Global Positioning System or GPS tracking compared to lower-level athletes (Fig. 1).

### Recovery modality

**Fig. 2:**
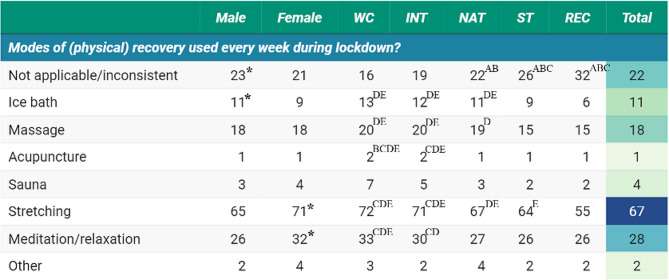
Recovery modes by gender, athlete level, and total cohort (percentage) during the COVID-19 lockdown *Note*: WC: World Class; INT: International; NAT: National; ST: State; REC: Recreational. *Significantly higher (or significant contributor to the relationship); superscript letters ^A, B,C, D,E^ represent significantly higher than World-Class, International, National, State, and Recreational levels, respectively

Stretching (67%) was considered the primary modality for recovery, especially by the higher-level athletes (World-Class 72%, International 71%) as well as female (71%) athletes. These modalities were followed by meditation (28%) and massage (18%); which favored higher-level athletes (World-Class and International) (Fig. 2).

### Sleep pattern

**Fig. 3:**
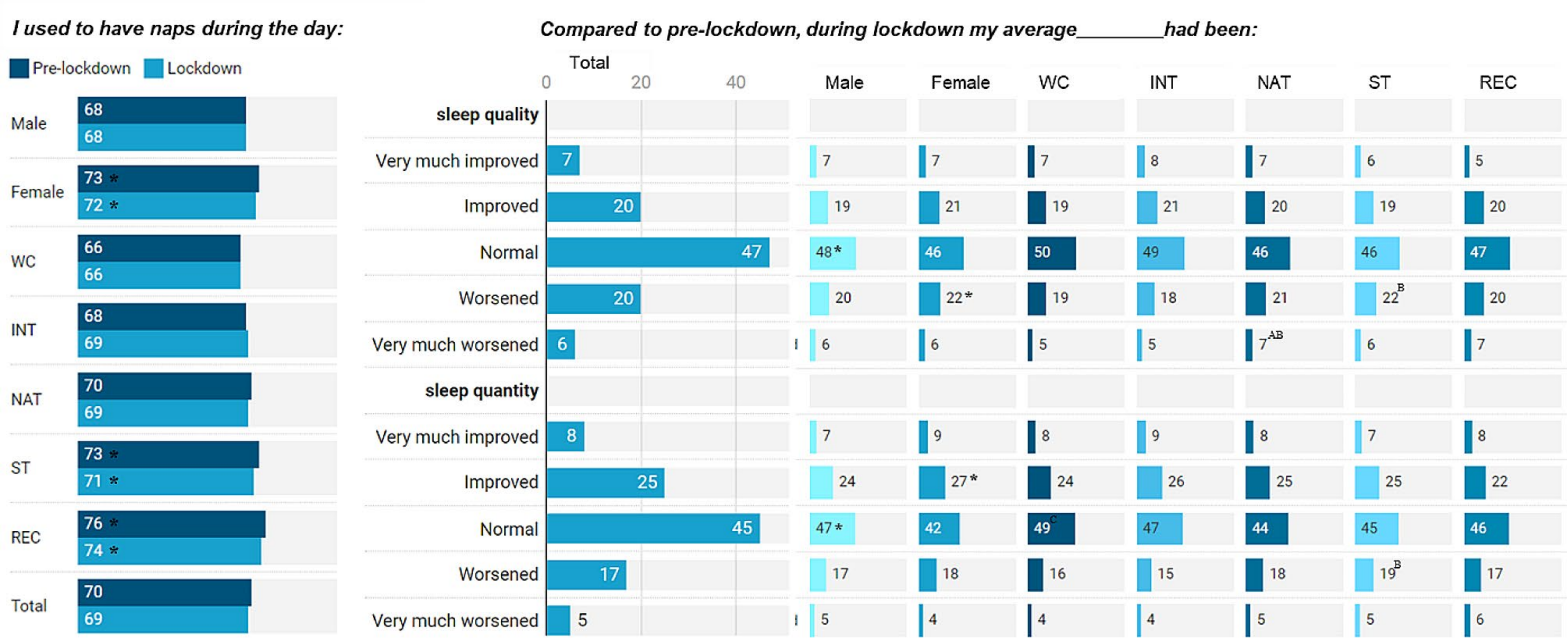
Sleep pattern based on gender, athlete level, and total cohort (percentage) during the COVID-19 lockdown *Note*: WC: World Class; INT: International; NAT: National; ST: State; REC: Recreational. *Significantly higher (or significant contributor to the relationship); superscript letters ^A, B,C, D,E^ represent significantly higher than World-Class, International, National, State, and Recreational levels, respectively

Napping practices before (70%) and during (69%) lockdown were generally similar. Nevertheless, marginally more Recreational and State athletes (71–76%) took naps than World-Class athletes (66%). Compared to pre-lockdown, about two-thirds of athletes reported “normal” or “improved” sleep quality and quantity (Fig. 3).

### Musculoskeletal injury and injury prevention

**Fig. 4:**
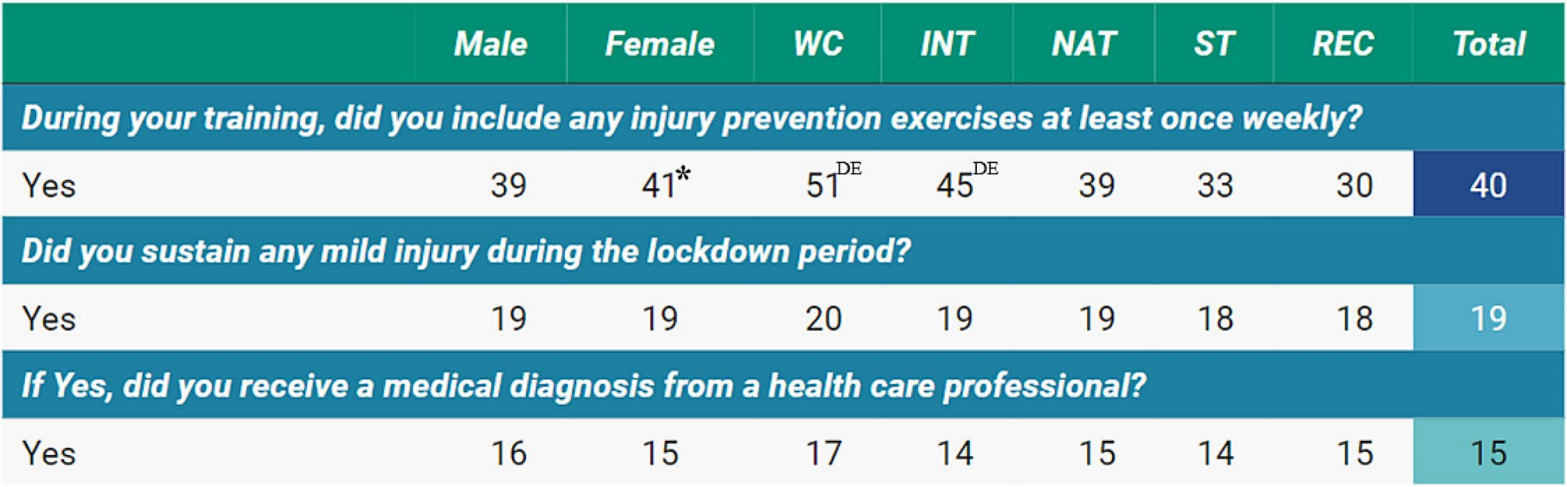
Musculoskeletal injury by gender, athlete level, and total cohort (percentage) during the COVID-19 lockdown *Note*: WC: World Class; INT: International; NAT: National; ST: State; REC: Recreational. *Significantly higher (or significant contributor to the relationship); superscript letters ^A, B,C, D,E^ represent significantly higher than World-Class, International, National, State, and Recreational levels, respectively

During lockdown, injury prevention exercises once a week were undertaken by 40% of the athletes. More World-Class (51%) and International (39%) athletes completed these prevention strategies than State (33%) and Recreational (30%). Injury prevention was applied by following either dedicated programs (FIFA11+, HarmoKnee, VolleyVeilig, SportsMetrics, KIPP) or by certain modalities such as neuromuscular and specific exercises (Nordic hamstring, Copenhagen, jumping lunge, etc.), mobility (hips, ankle, elbow, pelvic, etc.) or multimodal exercises (flexibility + strengthening + balance + jumping). 19% of athletes experienced a minimal or mild injury (1 to 7 days lost from training). 15% of the injured athletes received a medical diagnosis from a healthcare practitioner (Fig. 4). Most of the injuries involved the knee (18%), ankle (16%), and back (9%).

### Overall home training experience

In a qualitative evaluation of one open-ended question, four key themes emerged regarding athletes lockdown experiences (Table 1): (i) adaptation to remote training; (ii) creativity in training practices; (iii) performance enhancement opportunity; and (iv) mental and motivational challenges. In addition, athletes had various opinions about lockdown situations and its associated effects (Table 1). Table 2 (*at the end of article*) provides selected remarks concerning lockdown challenges and the experience of individual athletes from 45 countries.

**Table 1:** Summary of major themes related to home training experience

Themes	Sub-themes
Theme 1: Adaptation to remote training	o Finding solutions to challenges; adapting to situations, maintaining motivation, enthusiasm, and manage psychological issues. o Ensuring that training objectives (broadly) are still met. o Utilizing available resources (online, digital tools; purchase, borrow). o Shifting training focus to maximize resource availability.
Theme 2: Creativity in training practices	o Applying creative and alternative training approaches. o Utilizing available means (such as household items) creatively. o Modifying routines using unconventional or novel ways. o Applying a variety of training (innovatively) to meet fitness goals.
Theme 3: Performance enhancement opportunity	o Improving performance parameters previously neglected. o Enabling more focus on exercise routines. o Having extra time available for training, recovery, psychology. o Analysing technical aspects (such as running).
Theme 4: Mental and motivation challenges	o Difficult to stay motivated to train due to competition uncertainty. o Difficulty of training without competition objectives. o Social aspect of training with teammates.
Theme 5: Opinions related to lockdown situations and associated effects	o Inadequate support from organizations (sport sciences, financial, recovery). o Struggling with training due to limited equipment and spaces. o Monitoring of training by coaching staff, etc. o Negative impacts on overall health and finances. o Anxiety and uncertainty contributing to poor nutrition intake. o Prioritizing health over sports. o Planning for elite athletes (training bubbles).

**Table 2:** Selected remarks concerning lockdown challenges and experience of individual athletes from 45 countries

Country, gender, age	Sport (level)	Remarks
Albania, M, 50	Football (ST)	“Better training during confinement, with my family; we used simple exercises and programs which are fun”
Argentina, M, 31	Swimming (ST)	“It’s difficult for me to excel in open water swimming, so I chose to start triathlon; I have a roller to do quality (bike) training sessions”
Australia, M, 61	Cycling (ST)	“Lockdown is devastating for my health and financial situation. Our government has to stop this. I want my life back”
Australia, F, 29	Rugby (NAT)	“While my training intensity and duration has been more or less maintained, the lack of training partners is impacting on motivation and enjoyment”
Australia, M, 30	Cycling (INT)	“I have small injuries and needed to get adequate recovery, but I still keep training as I’m afraid to lose fitness if I quit training completely”
Belgium, F, 26	Swimming (ST)	“I used to do many of my sessions in the gym, both running, cycling and resistance training. With the lockdown I ran and cycled outside all the times”
Belgium, F, 28	Swimming (WC)	“I trained 20–22 hours weekly. Trying to find new routines; began at 7:00 am with cardio or stabilization, then 2–2:30 hours swimming and twice-weekly weight training. I cycled a lot and set biweekly challenges for motivation”
Benin, M, 30	Football (INT)	“Due to the closure of stadiums and sports facilities, my training sessions have been more jogging outdoors (9-15km / session).
Brazil, F, 23	Sailing (NAT)	“The biggest difference for me is training alone. I would usually run with training partners 4–5 times weekly. But I’m now able to add running sessions as I work from home; often with shorter sessions but run twice a day”
Brazil, M, 24	Taekwondo (NAT)	“I try to recall what I was doing during the training and I also attempt to change my daily habits. I try to follow my regular routines”
Brazil, M, 19	Judo (ST)	“Facing economic issues and could receive more support (needs assistance)”
Brazil, F, 26	Judo (WC)	“I bought training materials to practice at home, I did a small change in the bedroom in my parent’s house to have more space for training”
Canada, M, 51	Cycling (NAT)	“Not having access to gym facilities. I can no longer do the weight training that I do as a means of maintaining strength and preventing injuries.”
Canada, F, 24	Runner (NAT)	“It has been easy to continue with running training (minimal facilities); difficulty in maintaining motivation with resumption uncertainty, and S&C (no proper equipment at home); I suffered a mild quadriceps strain”
Canada, M, 52	Cycling (NAT)	“Have more time available, so my training and recovery quality have improved; no need to go to work or having any other social engagements”
Canada, M, 54	Cycling (INT)	“Enjoyed it, never ever had the opportunity to be with family for such a long time; I train twice a day at home, and being surrounded by my partner”
Canada, WC, 28	Biathlon (WC)	“I recommend that World sport bodies compel National bodies to make available training programs for their athletes should cases like this reoccur.”
Chile, F, 24	Hockey (ST)	“Initially, I ran on a treadmill, and now back on the road within a limited range; amazed how my glutes and hamstrings weakened over the time period”
Chile, F, 22	Wushu (NAT)	“I do not consciously feel anxious (but maybe subconsciously). Moved from more sport-specific training to a more general fitness, base-building, phase”
Chile, F, 26	Hockey (ST)	“Personally, with the anxiety and uncertainty about the situation, it has been difficult for me to maintain the diet and supplementation schedule.”
Chile, F, 23	Hockey (INT)	“It has all been progressive; I didn’t have any implements, but little by little I bought basic things, like bands, rope or dumbbells. A week ago the team gave me an Olympic bar with weights so I could train better”
Colombia, F, 27	Cycling (ST)	“It was hard staying motivated to train not knowing when I will compete again”
Croatia, M, 22	Karate (NAT)	“Reduced length of longer sessions; focused more with S&C work. Reduced intensity: mitigate being unwell and infection risk, and uncertainty to compete”
Croatia, M, 19	Athletics (INT)	“I trained as before the lockdown mostly because the conditions in my hometown are so poor that there was no difference”
Czech, F, 22	Swimming (INT)	“Typically most of my training sessions are in swimming pool, but I did not have access to the swimming pool during the COVID-19 lockdown”
England, M, 43	Cycling (INT)	“I am blessed to have the mental strength and coaching support”
England, F, 28	Triathlon (ST)	“My overall training volume has increased, but my swimming has gone down to zero. General fitness improved, swim-specific fitness dropped dramatically”
England, M, 33	Martial arts (INT)	“The lockdown was not good for our overall health mentally, physically, and financially”
Finland, F, 35	Bodybuilding (INT)	“I was able to get all the needed equipment at home, so my training was very versatile”
Finland, F, 29	Baseball (NAT)	“I am a team sport. I felt that training in small groups was irresponsible, thus I trained alone. Responsibility and clear instructions are needed”
Finland, F, 25	Football (WC)	“I am afraid of getting injured. Football season starts after 2 weeks of notice period that is not enough”
Finland, F, 25	Biathlon (WC)	“I usually travel to high altitude during summer for a training camp. If not able to do that that is going to have a huge impact on me”
France, M, 47	Tennis (NAT)	“Biggest limitations are motivation, loss of fitness, and weight gain. Unable to push harder; training 4-6hrs on the stationary bike drives me mentally insane”
France, M, 43	Biathlon (NAT)	“It’s possible to continue running (even outdoors), in cycling (on a home trainer), but not in swimming. So, maintaining physiological and technical capacities was possible on some of the activities”
France, F, 30	Athletics (NAT)	“The most difficult thing is to run without a competition objective. No specific work. Lack of sleep. Sport was a mental relief to go through confinement”
France, F, 27	Gymnastic (INT)	“Lockdown allowed me to realize how very important competitive sport is in my life. Now I am even more determined to give everything during my workouts, and every moment spent in the gym”
France, M, 58	Swimming (ST)	“I would disobey the confinement recommendations by training in a natural environment, lagoon plus mountain”
Germany, M, 49	Rowing (NAT)	“I am fortunate that I have a very good home gym set up so the lockdown did not affect my training. In fact my training has improved by working at home online (more energy to train as less energy was expended during the day)”
Greece, M, 19	Football (ST)	“I improved strength and flexibility, parameters that I neglected previously”
Hong Kong, M, 33	Football (INT)	“It’s very important to have a psychology (session) in this time, it will help a lot to keep your mind strong and focus on your future.”
India, M, 21	Football (ST)	“Stress from the pandemic severely impacted my motivation and ability to concentrate, which in turn impacted my training intensity and duration”
India, F, 19	Cricket (NAT)	“I’m struggling with training due to limited space for training. The restricted outdoor time is inconvenient, and unsafe (being outside) for me as a woman”
Indonesia, M, 23	Hockey (NAT)	“The athletes’ training program needs to be monitored by the coach no matter the condition is (social distancing)”
Indonesia, F, 19	Sport Climbing (NAT)	“The athletes need funding and facilities to maintain their (physical) conditions during the lockdown”
Indonesia, F, 31	Paragliding (NAT)	“It’s important to check athletes’ programs. Uploading videos on social media will help to boost the spirit to keep practice during the pandemic”
Iran, F, 32	Swimming (ST)	“Sports TV shows teaching of Taichi and Yoga, was very useful for me”
Ireland, M, 60	Cycling (NAT)	“I found that lockdown is beneficial to my training. Working from home allows me to better manage my training; I have more time to train. Traffic was less, it’s more pleasant to cycle. Overall it has been positive for me”
Luxembourg, F, 42	Triathlon (NAT)	“Stopped swimming during confinement but increases cycling training”
Luxembourg, M, 25	Handball (INT)	“As a team player, I missed seeing my teammates and the physical contact while playing handball”
Malaysia, M, 23	Badminton (ST)	“Injured before lockdown, I have problems in recovery or rehabilitation”
Malaysia, F, 18	Sport climbing (INT)	“It’s hard to maintain performance and even harder to improve; especially on the technical side. Physical can still be trained, but specific muscles for climbing cannot be developed or maintained without wall climbing”
Malaysia, M, 24	Wheelchair Tennis (INT)	“For elite athletes, to maintain their performance, ideally gather them in a safe and suitable place for training; provide food and medical facilities; plus mini games (PlayStation, snooker, or other indoor games) for them to release stress”
Malaysia, M, 19	Hockey (ST)	“I was not able to maintain my pre-lockdown fitness level. The longer this lockdown lasts, the less motivation I have to training and exercise”
Malaysia, M, 21	Archery (NAT)	“I do not have time to practice well because there are too many university online classes and assignments”
Malaysia, M, 21	Handball (NAT)	“Mental preparation is very important. Also, hopefully that there will be many more virtual applications created for specific or technical training”
Mauritius, M, 38	Athletics (WC)	“I found it very hard to motivate myself to train at all, with increased anxiety and disruption to routine being the biggest factors.”
Nepal, M, 21	Football (INT)	“Mental health is affected obviously by this lockdown but in the other hand relationship between family members can be improved and mostly with God”
New Caledonia, F, 32	Cross Country (INT)	“I was able to devote myself fully to physical activity (previously not possible); exploring and enhancing my physical and mental strengths, and found pleasure and balance during the confinement period”
New Caledonia, M, 43	Muay Thai (INT)	“Confinement was a perfect opportunity to do rigorous physical preparation without the stress of everyday life”
New Zealand, M, 45	Ironman (NAT)	“Training alone for months without competition has been tough. Felt nervous, I can’t train with anyone in my family (others have siblings). Others might be ahead of me and how that might affect my abilities when we do return to play”
New Zealand, M, 32	Judo (ST)	“Lockdown has only impacted endurance. My average cycling was 16.5hr/wk, now it is ∼ 8–9 hrs/wk. Light weight lifting at home has increased”
New Zealand, F, 19	Rugby (INT)	“I purchased a smart trainer just prior to lockdown starting, knowing that outdoor cycling would become a criminal offence”
Norway, F, 21	Taekwondo (INT)	“I have periodically lost motivation for specific training and have therefore seek other challenges for physical and mental conditioning”
Norway, F, 20	Boxing (INT)	“As an amateur athlete, I have been left alone over longer periods. Challenging to practice during such circumstances.”
Norway, M,	Nordic Combined (WC)	“I have partly taken a time off after the season”
Norway, M, 24	Canoeing (WC)	“One extra year without money for training is killing me economically”
Pakistan, M, 41	Cricket (ST)	“I found that not having access to rehabilitation facilities has been my frustration during the lockdown”
Peru, M, 23	Athletics (INT)	“No support from government institutions during lockdown”
Philippines, M, 26	Triathlon (INT)	“Training and in particular racing on Zwift (virtual cycling / running world) helped me a lot to stay motivated; I couldn’t do any swim training at all”
Philippines, F, 20	Basketball (ST)	“Maintain a positive outlook, healthy habits, and following safety guidelines protects against COVID-19, and supports our well-being. Despite the challenges, spiritual growth helps us stay resilient and connected with God”
Philippines, F, 19	Volleyball (NAT)	“Decided to lower exercise volume and intensity for better health and less immune system stress; aiming for 1 hour daily with a rest day”
Romania, F, 22	Handball (NAT)	“Every athlete has likely faced physical and mental challenges, leading to stress and frustration. I recommend post-lockdown meetings with a psychologist for athletes and their core teams, including coaches, for mental well-being”
Russia, F, 20	Shooting (NAT)	“To combat viruses, strong immunity from fresh air, exercise, and good nutrition is key. Isolation weakens immunity. Remote coaching doesn’t help; we need competition, communication, and energy exchange; impossible now”
Scotland, M, 59	Triathlon (INT)	“My main challenge has been a lack of swim time. Cycling is OK (with Turbo). I need a consistent training for my fitness and technique; if competitions resume, it will be tough, though not impossible”
Singapore, M, 21	Ultimate Frisbee (INT)	“Lack of competitions soon anywhere may have restricted strategy towards planning training blocks”
Singapore, F, 35	Cycling (INT)	“Online bike racing instead of real life allowed more frequent and sometimes more intense efforts”
Slovenia, M, 25	Handball (INT)	“Lack of sports science support”
Slovenia, F, 22	Judo (INT)	“I believe that the quarantine will leave long-lasting consequences for all athletes”
South Africa, F, 31	Athletics (NAT)	“Health is a priority not sports. It’s our duty to help others as we are more fit than others. Lockdown is important. Sport is not just medals, it’s for happiness. For some people’s, it’s business”
South Africa, M, 25	Hockey (INT)	“I believe that more education regarding training is required”
South Africa, M, 40	Rugby (NAT)	“Lockdown enabled me to focus on exercise routine like never before. Mental balance is vital. (Every day) I always had a (training) goal in mind”
South Africa, F, 27	Netball (ST)	“Had a tendency to over train during lockdown. Had to monitor closely”
South Africa, M,	Athletics (INT)	“I was able to do speed, speed endurance, and general fitness training (running virtual races); but, unable to do hurdles, jump technique, and throws”
Spain, M, 50	Aquatic (INT)	“I think strict lockdown prohibiting daily walks or run will finally kill or affect more people than the COVID-19 itself (physical and mental health! )”
Spain, M, 39	Athletics (ST)	“During lockdown, my typical workouts are: 3 days of strength training, two of HIIT and one of light aerobic exercises”
Spain, M, 18	Swimming (NAT)	“My sport swimming, it is very difficult to maintain fitness since we do not have the means (pool) to practice the sport”
Sri Lanka, M, 45	Shooting (WC)	“Officials from all sporting organisations need to follow up on all individuals who are involved in their organisations during troubled times”
Switzerland, F, 21	Athletics (NAT)	“I maintained my usual training volume during the first phase of the lockdown (3 weeks), then I greatly reduced the quality and quantity of training when I learned of the cancellation of competitions”
Tunisia, M, 21	Sailing (WC)	“Lockdown didn’t harm my mental health, in fact, benefited my physical well-being, as it gave me a break from studies and more time to devote myself psychologically and physically”
Tunisia, M, 40	Football (NAT)	“I think the big concern is not training alone during confinement, but the fear of injuries caused by the excess load”
Turkey M, 20	Wrestling (NAT)	“I want to start training again. If the lockdown period prolongs it will cause further injuries when we start training”
United States, M, 18	Swimming (NAT)	“I missed my teammates and the motivation that comes along with training alongside them. I’m so sick of my stationary bike”
United States, M, 20	Athletics (ST)	“The loneliness experienced during the lockdown made me more motivated in achieving my future goals”
United States, M, 21	Cross Country (NAT)	“Good survey… athletes and coaches should take a lesson, ensuring basic equipment readiness for any scenario; adjust training. No more complaints, just work and enjoy all sessions. Cultivate a mind-set to find positivity in adversity”
United States, M, 19	American Football (INT)	“Lifting weights in a friend’s garage was essential for maintaining my routine; I have reasonable options. Closed tracks made interval training difficult; but I adjusted my regimen. Adapting mentally to this new routine was challenging”
United States, F, 52	Cycling (INT)	“I improved my running by studying my technique on a treadmill with video analysis. This was ideal timing as I was returning from an injury, allowing me to rebuild my running economy and cadence from scratch”
United States, M, 20	American Football (INT)	“I worked on improving my running economy and stride frequency through video analysis (on treadmill) during my recovery from injury and lockdown”
United States, F, 20	Athletics (WC)	“It’s challenging for unsponsored athletes to maintain communication with coaches due to high data costs, impacting their training. Access for massage and physio was limited; hinders effective recovery; it’s frustrated”
United States, F, 26	Athletics (WC)	“Training during lockdown was a motivation to improve your capacity to climb the difficult moment and has a very positive impact on our mentally. This period was a lesson and it will help us to be stronger than any time before”
Uruguay, M, 31	Triathlon (NAT)	“My big absence in training was swimming. Previously, I didn’t do strength exercises and now I’m doing it twice a week”

Note: Athletes’ remarks had been edited for language; WC, World Class; INT, International; NAT, National; ST, State; REC, Recreational

## Discussion

This is the first global survey investigating athletes’ alternative training, monitoring, recovery modality, injury/injury prevention, and sleep patterns during the early COVID-19 lockdown. Athletes from various countries, across different sports and competition levels, adapted to these unprecedented circumstances in different ways. About a quarter of athletes, predominantly at higher levels, implemented alternative training methods, including virtual reality and improvised equipment. Interaction between athletes and coaches was not frequent, with higher-level athletes more actively monitoring training loads (facilitated by their coaching staff). Physical recovery strategies were dominated by stretching (67%), but some athletes also considered meditation, which was more prevalent among higher-level athletes. Sleep patterns showed little change, though higher-level athletes reported fewer naps. While training during lockdown, injury prevention exercises were also incorporated (40%), particularly among World-Class and International athletes. Analysis of open-ended responses revealed four major themes related to overall home training experience: (i) remote training adaptation, (ii) training creativity, (iii) the opportunity to enhance performance, and (iv) mental and motivation challenges. These insights demonstrate the adaptability of athletes across different competitive levels with changes and shifts they made during the COVID-19 lockdown.

Many athletes employed creative solutions to overcome lockdown restrictions. Notably, home-based exercise equipment (e.g., smart bikes) combined with virtual reality technology (e.g., Zwift) connects individuals remotely, offering interactive and realistic exercise scenarios; contrasting with typical lockdown isolation [26]. Importantly, some athletes reported using a “more traditional” or standard equipment/tools (e.g., treadmill, roller bike, mini gymnasium, or a swimming pool) [27, 28]. Home-based training has yielded inconsistent results in different fitness components [7, 29]. Regardless of the options employed, it is crucial to regulate training variables (intensity, duration, frequency, etc.) to minimize the loss of neuromuscular adaptations [30] and preserve fitness levels [1, 31]. A country-level study revealed that athletes, particularly the World-Class cohort, maintained their weekly training frequency despite compromise in other key training variables, such as duration and intensity [10]. Online coaching has become crucial in this context, with the emerging role of technology being indispensable in maintaining effective training routines under “difficult” conditions.

Effective training during lockdown necessitates regular athlete-coach communication to enhance adherence to remote programs and the interaction/feedback process [3]. This, in turn, enables effective monitoring of progress and training loads. The latter aids in ensuring desired effects on athlete well-being and performance [32], while minimizing the risk of developing non-functional overreaching, illness, and/or injury [33]. Therefore, enhancing the frequency of communication between coaches and athletes is essential, particularly considering the observed low rates of daily (22%) and weekly (36%) communication between athletes and coaches during lockdown, especially for lower-level athletes. It is plausible that the nature of coach-athlete interaction varies by sport; likely more often in higher-level team sports (for team training), but potentially less in individual sports (athletics, cycling, etc.), where the coach’s role might lean more towards an advisory capacity for athletes. In the context of load monitoring, there is a notable variance across athlete levels, with a greater engagement among higher-level athletes (≥ 54%), compared to State and Recreational levels (< 40%). Coaches were more involved in load monitoring at higher-levels (Fig. 1), predominantly using heart rate monitors (21%) and daily diaries (19%), although minimally. Objective monitoring (e.g., heart rate monitors) offers information that allows for immediate adjustments to training intensity, while subjective measures (e.g., rate of perceived exetrion, diaries) can track the perception of effort and training responses (including internal/external loads) such as training volume, muscle soreness, and mood [32, 33]. These practices suggest a trend towards more practical monitoring methods for pandemic home training among athletes. Nevertheless, adoption of proper home-based training protocols, including training load, remains pivotal to avoid a delayed or unsafe return to sport [30], irrespective of athlete levels.

Athletes implemented a variety of recovery methods, including stretching, meditation, and massage. This observation reflects a growing awareness of the importance of recovery and the ability to pivot to accessible, self-administrable methods amid lockdown constraints. Post-exercise stretching (cool down) is commonly prescribed to enhance recovery and alleviate delayed onset muscular soreness after physical exertion [34]. Moreover, meditation has been shown to positively impact athletes’ mental skills and performance, with various techniques linked to enhancements in movement, physical health, and mental well-being [35]. While massage is a widely utilized recovery practice among athletes, its implementation was reduced to less than 20% during lockdown, likely due to access limitations (e.g., restricted movement for masseurs). Nonetheless, athletes might have resorted to self-massage techniques (e.g., foam rolling), sought assistance from housemates, or utilized automated massage devices (e.g. vibration tools). In the latter instance, massaging tools such as a massage gun are easy to use and appear effective in improving recovery-related outcomes [36].

Central to these recovery methods is recognizing sleep as a critical component to maximize recovery (physiological and psychological) from training [37]. Notably, two-thirds of athletes report normal or improved sleep quality (during lockdown), with napping habits remaining consistent pre- and during-lockdown. Despite this, some athletes increased daytime napping after reduced nocturnal sleep quality and higher insomnia, which can affect homeostatic sleep regulation and performance [17]. Interestingly, maintaining high training intensity during-lockdown was associated with better sleep quality [38] in elite athletes, and vice versa [39]. In line with the current finding, elite athletes often report modest sleep quality pre-lockdown [40] likely a consequence of demanding schedules, such as training, travel, and media obligations, which were “limited/alleviated” during lockdown. Key practices for improved sleep quality include avoiding long and late daytime naps, avoiding caffeine consumption in the second half of the day while maintaining regular meals, training, and sleep schedules [17]. Athletes would benefit from improved sleep hygiene, such as establishing regular evening or pre-sleep routines, consistent wake-up time, and creating an optimal sleep environment [41].

Many athletes (40%) engaged in injury prevention measures, with greater practices in World- Class and International athletes than at other levels. The occurrence of injuries (most commonly, knee, ankle, and back injuries) during lockdown highlights the need for safe, regimented training within limited resources. Previously, it has been reported that two-thirds of injuries during lockdown affected the lower extremities [42]. In addition, increased daily sitting duration [43] may be one possible cause of low back pain [44]. Moreover, most injuries (67%) occurred during the first week after the start of lockdown [42]. It is plausible that home-based injury prevention training may have lacked customization to athletes’ specific needs. Individualized injury prevention programs are best identified through targeted fitness assessments (pre-season; existing data), including neuromuscular and biomechanical evaluations (among others) [3]. Furthermore, athletes might have been inadequately prepared for post-lockdown training demands, increasing the likelihood of injuries [42]. Injury may also be attributed to a reduced long-term training load and a sudden increase in training intensity following the lockdown, which increased injury risk [13, 45]. These risks highlight the importance of injury prevention practices as well as proper training load management during (and post) lockdown. Training volume and intensity compliance also do not exempt athletes from factors that may lead to musculoskeletal injuries [42]. Psychosocial elements and observed emotional behaviors during lockdown, may also contribute to injury risk beyond physical fitness/factors [15].

Despite facing multifaceted lockdown challenges (e.g., mental and motivation), some athletes identified ways to adapt to remote training, demonstrated creativity in training practices, and viewed the period as an opportunity to enhance performance (Table 1). Given the lockdown restrictions, athletes often trained alone, thus without adequate supervision [1]. Athletes displayed symptoms (e.g., signs of mental health issues) and disorders at levels comparable to or even surpassing those in the general population [20] possibly related to potential injuries, loss of income or sponsorships, competition uncertainty, and fears of disease (Table 2). Within this context, elite athletes sought assistance in performance lifestyle for managing (supporting) their careers [5]. Interestingly, elite athletes, who had better access to support, demonstrated a notably higher resilience to disruptions caused by the pandemic [46]. From the psychosocial perspective, athletes’ mental health is affected by social support, psychological safety, communication, and self-behaviors [47]; thus, the involvement of family members (i.e., parents and siblings), coaches, teammates, may play a substantial role in regulating or reducing stress [47]. Likewise, keeping athletes informed about available psychological-related services (screening, consultation, etc.) is crucial in promoting a proactive environment for their mental well-being [48].

Some athletes in this international cohort seized the “opportunity” of lockdown to focus on aspects they neglected previously or were simply inaccessible, such as (i) recording and analyzing running technique, (ii) engaging in rigorous physical preparation without everyday life stress, (iii) allotting more time for training and recovery quality due to reduced social engagements and work, (iv) focusing on important but previously neglected training (e.g., muscular strength and flexibility for endurance athletes), and (v) enjoying training with a partner and family at home, or even allow more prolonged periods of rest/sleep (Table 2). Despite the challenges, lockdown proved to be a “blessing in disguise” for some athletes, as noted above. The ability to effectively respond to challenges and mitigate the potential negative effects of adverse experiences may be seen as protective factors, e.g. resilience of individuals and self-regulation [49]. Furthermore, motivation toward training is vital for continuity and adherence [50], while training disruption could influence willingness to train [51]. During lockdown, athletes faced “difficulty maintaining motivation due to resumption uncertainty,” among others (Table 2). Motivation is influenced by autonomy (ability to choose training activities), competence (desire to produce desired results), and relationships (feeling of being connected with others) [52]. The roles of a network of coaching staff, friends, and family support (including team environment) are crucial for maintaining emotional well-being and motivation [53]. Indeed, the absence of a “social facilitator” and team interaction can markedly impact athletes’ motivation and decision-making in sports [54, 55]. As such, changing to a (favorable) training environment (e.g., bubble training) would also help regain athletes’ motivation [5]. Moreover, athletes who followed training programs, either designed by coaching staff or sourced externally, demonstrated reduced anxiety and a higher level of motivation to return to sport upon resumption [56].

This study highlights a comprehensive insight into performance-related challenges and coping strategies athletes worldwide adopted during the COVID-19 lockdowns while accounting for potential moderating factors such as the participants’ gender and experience levels. A major strength of this study lies in its large sample size that covers a wide range of athletes’ routines and challenges. To the best of our knowledge, this research is among the first to document the varied challenges and difficulties faced by athletes from numerous countries in both pre-set and unstructured question-and-answer formats. Nevertheless, it is crucial to acknowledge some limitations that should be considered when interpreting the present findings. The study’s cross-sectional design limits our ability to infer causality. Additionally, the reliance on self-reported data may introduce recall and subjective biases. Importantly, the injury findings pertain exclusively to athletes with “mild injuries,” as those with moderate or severe injuries did not meet the inclusion criteria. Various degrees of lockdown existed across the world. However, we only considered countries and territories that implemented a “medium to high” lockdown severity (at least). Lockdown coinciding with Ramadan fasting could have influenced the results [57], which we further addressed elsewhere [58]. Finally, it is also important to keep in mind that while the unique context of the COVID-19 pandemic might affect how broadly the findings or recommendations can be applied, they nonetheless provides valuable lessons that could be applicable to other challenges, such as a natural disaster, geopolitical or religious restrictions, or government directives that could impact athletes regular training routines. Our recommendations may be more relevant with the interests and needs of athletes who do not have disabilities based on the demographic composition of our survey respondents. Future investigations could explore the policy implications for national federations, practical guidelines for coaches and athletes, further development of digital training tools, and more detailed analysis of how training can be modified when access to specialist facilities and equipment is limited.

### Recommendations for supporting athletes during challenging contexts

In this study, we characterized and discussed factors that have the potential to impact an athlete’s performance during “challenging contexts.” Our analysis serves as the foundation for generating informed recommendations. First of all, sports organizations should provide athletes with comprehensive guidelines for navigating extraordinary circumstances such as pandemics, curfews, pollution spikes, extreme weather, and social crises. Next, it is imperative to prioritize the welfare of athletes, ensuring any potential risks or harms are addressed promptly, with their well-being taking precedence over training considerations. As we focus on athlete’s performance and training, we recommend consultation of specific guidelines that address general health, health risks, and necessary screening pertinent to exceptional circumstances (e.g., COVID-19). Also, we are aware of the usefulness (and shortcomings) of AI conversational tools (e.g., ChatGPT) in providing support/prescription for training, mental, nutritional, education (among others) [59, 60], but these are beyond our current discussion’s scope. The following recommendations offer a balanced approach to maintaining physical and wellbeing during “challenging situations” (Table 3). While these recommendations are designed to be adaptable, they should be tailored to accommodate personal requirements for optimal benefits.

**Table 3:** Summary of recommendations

Alternative training	• Enable athletes to meet the targeted training objectives (e.g., strength, power, endurance, cardiorespiratory) and, if possible, perform exercises resembling sports movements (e.g., agility, short sprint, jumping, throwing). • Creatively using available means with unconventional methods (e.g., household items) in training routines can diversify workouts, and achieve fitness goals. • Athletes can be provided with either basic and/or specialized training. Some athletes may already possess standard equipment or tools such as a treadmill, a roller bike, a mini gymnasium, or a swimming pool. • Virtual training and competitions are accessible to many athletes. For example, virtual reality racing or fitness games that simulate riding, boxing or dancing (or even competition) can offer a fun and interactive training (and competitions). • The training should be periodized (proper progression, recovery, individualized, and monitored).
Training monitoring	• Insufficient training stimuli for extended periods may reduce the fitness of athletes. Fitness tracking applications such as Strava™ and MyFitnessPal™ allowed athletes to log exercises and monitor progress. An app like TeamBuildr™ allows coaches or trainers to provide daily workouts and receive athlete data feedback. • Scheduled video calls (daily or weekly) for training can be helpful for both athletes and coaches to anticipate meetings (online), discuss progress and challenges, and make any adjustments needed in training routines.
Injury prevention	• The issue of applying injury prevention training, particularly if resources are limited (home-based training), is lacking specificity. Ideally, injury prevention programs are tailored to the athlete’s specific needs. This is usually identified via fitness testing incorporating, among others, neuromuscular and biomechanical assessments. If this is not available, general prevention programs based on common risks associated with sports may be applied. • For optimal benefits, athletes need to be provided with appropriate programs by (qualified) coaches or trainers. Common injury prevention programs that may be adopted (fully or partially), can include multimodal exercises (e.g., flexibility + strengthening + balance + jumping + change direction), such as FIFA11+™. Applying a comprehensive, evidence-based program (e.g., NetballSmart™) can be helpful. Insufficient training and a sudden spike in intensity upon resumption may increase the risk of injury.
Recovery strategies	• Recovery is a broad term for restoring balance after physical or mental exertion. Recovery should be prescribed by considering the nature of training and load monitoring. • Various recovery modalities may be accessible (e.g., hot water, cold water, foam rolling, massage gun) by athletes. Sleep and nutrition should be emphasized as playing important roles in the recovery process. Stretching (physical) and meditation (psychological) are “popular” recovery methods employed as part of daily or weekly training. Athletes can also consider using automated massage devices such as massage gun, or self-myofascial release techniques via a foam roller or roller massager.
Sleep and napping	• Good sleep hygiene is crucial for athletes, particularly during challenging situations (lockdown etc.). Key practices include avoiding long (> 30 min) and/or late (after 16h00) daytime naps and limiting caffeine intake except early in the day while abstaining from alcohol and nicotine. • Maintaining regular meal, training and sleep schedules, and avoiding heavy meals near bedtime potentially enhances sleep quality. Establish a consistent, and regular sleep routines, including pre-sleep routines (e.g., reading, warm bath), wake-up time (everyday), aiming for 7–9 h each night. Create a comfortable sleep environment (e.g., lighting, temperature, ‘privacy’) free of distractions. • Training sessions can be implemented in the early part of the day, which aligns the internal clock with the natural sleep-wake cycle, to ensure better sleep quality and overall physical and psychological well-being for athletes.
Mental health and wellbeing	• Restriction situations forced athletes to train at home, usually alone, and often unsupervised. The aim should be to “prevent” psychological distress during their transition back to normal training and competitions. Anxiety and stress reactions may be exacerbated when injuries occur during lockdowns, contributed by factors such as loss of income or sponsorship, uncertainty about return to competitions, and anxiety about contracting disease, among others. • Multidisciplinary interventions (e.g., psychological, psychosocial) or assistance can be implemented to identify stressors and help athletes cope with challenges and continue their training and careers. Use of relevant tools (e.g., questionnaires) to screen for risk factors is suggested. • Use of communication technologies (e.g., videoconferencing and telehealth care) is recommended, and if athletes feel overwhelmed, they can reach out to a mental health professional for online therapy sessions. Inform the athletes about the possibility and availability of assistance when needed.
Training motivation	• Motivation toward training is vital for continuity and adherence. Staying connected with support systems including coaches, teammates, family, and friends is crucial for emotional support and motivation, helping athletes to remain balanced and prepared for when normal training and competition resume. • Virtual training sessions with coaches and teammates via video calls or videoconferencing can provide a sense of community and accountability. • Athletes feel less anxious and more eager to return to their sport if following structured training plans. Shifting to a “better” training environment, such as “bubble training”, can boost motivation levels.

## Conclusions

During the COVID-19 lockdown, athletes across various levels were able to employ a variety of strategies to maintain training activities. Many athletes, particularly those at higher-level (negligible differences in gender), incorporated alternative training methods such as home-based modified- and interactive (virtual reality) training. A key factor in maintaining effective training regimes was consistent coach-athlete communication, which facilitated the prescription and supervision of training loads and workload monitoring. Due to lockdown restrictions, athletes considered stretching as a means of recovery strategy while some athletes practiced meditation. Remarkably, higher-level athletes seemed to have “better” sleep patterns while prioritizing injury prevention measures. During home training, some athletes demonstrated adaptability (e.g., finding solutions to meet training objectives), creativity (use of household items), and even taking advantage of lockdowns to address previous training deficiencies. The present findings also highlighted the diverse coping strategies athletes employed, demonstrating their adept capacity to overcome the challenges posed by the COVID-19 pandemic and lockdown.

## Data Availability

The datasets used and/or analyzed during the current study are available from the corresponding author on reasonable request.
